# Understanding the effects of supervisory and coworker safety communication on construction workers' behavior

**DOI:** 10.3389/fpubh.2025.1660513

**Published:** 2025-08-20

**Authors:** Sainan Lyu, Jiade Xi, Peng Cui, Rita Peihua Zhang, Xiaoyan Jiang, Beibei Zhang

**Affiliations:** ^1^School of Civil Engineering, Hefei University of Technology, Hefei, Anhui, China; ^2^School of Civil Engineering, Nanjing Forestry University, Nanjing, China; ^3^Department of Applied Physics and Electronics, Umea University, Umeå, Sweden; ^4^School of Property, Construction and Project Management, RMIT University, Melbourne, VIC, Australia; ^5^School of Economics and Management, Anhui Jianzhu University, Hefei, Anhui, China

**Keywords:** supervisory safety communication, coworker safety communication, safety behavior, safety knowledge self-efficacy, safety motivation, psychological ownership for safety, construction workers

## Abstract

On high-risk construction sites, frontline workers are constantly exposed to dual channels of safety communication: supervisory safety communication (SSC) and coworker safety communication (CSC). While existing research has emphasized the general importance of safety communication in promoting safety performance, the differentiated effects and psychological mechanisms of SSC and CSC remain underexplored. To address this gap, this study aims to adopt a Conservation of Resources (COR) theory framework to examine how SSC and CSC influence construction workers' safety behavior (SB) through psychological mechanisms, and how these effects vary under different levels of work pressure (WP). A survey was conducted with 359 frontline construction workers in large-scale projects across China, and data were analyzed by multiple linear regression and an interaction analysis with simple slopes. Results show that SSC (β = 0.234, *p* < 0.001) and CSC (β = 0.545, *p* < 0.001) both positively affect SB. Mediation analysis confirmed the roles of SKSE, SM, and POS, with SM showing the strongest effect (β = 0.235, *p* < 0.001). WP was found to weaken SSC's effects but not CSC's. These findings advance COR theory by clarifying psychological resource pathways in safety communication. Practically, the study suggests differentiated strategies for leveraging supervisory and coworker communication to enhance safety under varying work pressures.

## 1 Introduction

The construction industry remains one of the most hazardous sectors globally, with persistently high rates of accidents and fatalities (1). Over 80% of construction-related incidents are attributed to unsafe behaviors (2), underscoring the need to explore the behavioral, psychological, and social factors that influence construction workers' safety behavior (SB) (3–5). Among these antecedents, safety communication has gained increasing attention, which refers to the exchange of safety-related information, instructions, and feedback among workers, supervisors, and other stakeholders to promote safe practices and prevent accidents (6, 7). Safety communication has been regarded as a key dimension of safety climate in the construction sector (8, 9). Empirical studies have shown that effective safety communication not only enhances safety participation and compliance but also reduces cognitive failure, psychological stress, and injury rates among workers (3, 10–12). Its quality, frequency, and style significantly differentiate high- from low-safety-performing teams (12–14). These findings highlight the foundational role of safety communication in driving safety outcomes.

Despite the recognized importance, prior research often treats safety communication as a unified construct, overlooking the critical distinction between supervisory safety communication (SSC) and coworker safety communication (CSC). SSC typically reflects mostly formal, vertical communication emphasizing compliance and accountability, whereas CSC represents mostly informal, peer-level interaction grounded in daily collaboration and shared experiences (13–15). The limited research on SSC primarily focuses on its role in enhancing safety climate, which then indirectly shapes individual SB (10, 16). While safety communication is often embedded within discussions of safety climate, the two constructs are distinct. Safety climate reflects workers' shared perceptions of organizational safety priorities and practices, whereas safety communication pertains to the actual exchanges of safety information and feedback (17). In contrast, CSC has received less attention in terms of its underlying mechanisms and effects on safety outcomes. From a practical perspective, failing to distinguish SSC and CSC hinders the design of tailored communication strategies, while theoretically, it restricts a deeper understanding of how different safety communication sources drive safety outcomes. Thus, it is essential to disentangle the distinct roles of SSC and CSC in shaping SB, and to examine the psychological mechanisms through which each operates.

This study draws on the Conservation of Resources (COR) theory, which views individuals as resource-conserving agents striving to acquire, maintain, and protect valued resources under stress. Within this framework, psychological resources—such as safety knowledge self-efficacy (SKSE), safety motivation (SM), and psychological ownership for safety (POS)—are considered essential drivers of SB. These internal resources enable workers to understand safety protocols, stay motivated to follow them, and take ownership of collective safety goals (6, 18, 19). SSC and CSC can serve as key resource-enabling mechanisms by fostering these psychological states. Through information exchange, emotional support, and peer reinforcement, safety communication helps build workers' confidence, motivation, and sense of responsibility for safe behavior. However, under high work pressure (WP) (i.e., a prominent stressor in the construction industry), these mechanisms may be disrupted, weakening the positive effects of communication. WP thus operates as a condition of resource depletion, potentially moderating both the direct and indirect relationships between SSC/CSC and SB.

To address these theoretical and practical gaps, this study aims to examine how SSC and CSC influence construction workers' SB through the mediating roles of SKSE, SM, and POS, and how these pathways are moderated by WP. Although safety communication may originate from multiple organizational levels, this study focuses on SSC and CSC as the two most proximal and frequent sources of safety-related interaction for frontline workers. To achieve this aim, three specific research objectives (*RO*s) have been proposed:

*RO*1: To examine the direct effects of SSC and CSC on SB of construction workers;
*RO*2: To investigate the mediating roles of SKSE, SM, and POS in the relationships between SSC, CSC, and SB;
*RO*3: To assess the moderating role of WP in the direct and indirect effects of SSC and CSC on SB.

Data were collected through structured questionnaires administered to frontline construction workers engaged in large-scale infrastructure projects in China. The use of first-hand field data ensures the empirical relevance and contextual validity of the findings, particularly in high-risk and high-pressure construction environments. To empirically test the proposed *RO*s, quantitative methods were employed, including multiple linear regression analysis (for RO1), three-step regression approach (for RO2), and moderated path analysis with simple slope testing (for RO3). This multi-method analytical strategy enables a robust examination of how different safety communication sources influence SB via distinct psychological pathways, while accounting for the boundary condition imposed by WP (20, 21).

Theoretically, this study contributes to literature by offering an integrated, dual-path model of safety communication, distinguishing the roles of SSC and CSC and uncovering three distinct psychological mechanisms through which they operate. It also extends COR theory by demonstrating how WP may weaken the resource-enabling effect of safety communication, advancing our understanding of boundary conditions in safety behavior models. Practically, the study provides evidence-based insights for designing differentiated safety communication strategies, encouraging safety managers to leverage both formal and informal channels tailored to workers' psychological resource needs—particularly in high-stress construction settings.

## 2 Literature review and hypothesis development

### 2.1 SSC and CSC as a social resource

From the perspective of COR theory, individuals strive to obtain, retain, and protect resources that are instrumental in achieving valued goals. In the high-risk environment of construction sites, safety communication goes beyond an information exchange process, but also a form of social resource embedded in daily interactions (22). It involves the dynamic exchange of safety-related information between individuals or groups to ensure that information regarding hazards, risks, and safety procedures is not only shared, but clearly understood and actionable (23, 24).

A growing body of literature across sectors highlights the importance of safety communication in safety-related outcomes. For instance, Michael et al.'s (85) found that effective communication between supervisors and subordinates significantly reduced recordable injuries in manufacturing settings. In the construction sector, safety communication has been found to affect workers' psychological states, safety climate, safety behaviors, safety performance, and even physical wellbeing (13, 14, 25–27). However, safety communication is not a homogeneous construct. Instead, it manifests through distinct relational pathways shaped by organizational structure and the nature of frontline work (28). Thus, scholars have recently differentiated between *supervisory safety communication* (SSC) and *coworker safety communication* (CSC), highlighting that safety messages flow both vertically (supervisor–subordinate) and horizontally (peer–peer). Table 1 presents representative definitions of SSC and CSC from the literature.

**Table 1:** Definitions and descriptions of SSC and CSC.

**References**	**Definition and descriptions**
Huang et al. (15)	A: Subordinates' perceptions of the extent to which their supervisor provides them with relevant safety information about their job (i.e., top-down communication) and the extent to which they feel comfortable discussing safety issues with their supervisor (i.e., bottom-up communication). B: ——
Zhang et al. (10)	A: Two-way communication between supervisors and workers B: ——
Ishdorj et al. (6)	A:—— B: Worker-to-worker sharing of valuable safety knowledge
Zamani et al. (28)	A: It includes formal interactions with managers in the forms of safety training, work orders, written notifications, safety signs, and toolbox talks. B: The exchange and sharing of safety knowledge among members of a community in order to perform their tasks safely or to gain knowledge of risks
Cong et al. (33)	A: —— B: A worker may point out unsafe behaviors and remind co-workers about safety.

A = SSC; B = CSC.

SSC is defined as the extent to which subordinates perceive that their supervisors provide them with safety-related information regarding their work (i.e., top-down communication), and the extent to which they feel comfortable discussing safety issues with their supervisors (i.e., bottom-up communication) (15). SSC includes formal channels, e.g., safety meetings, briefings, and toolbox talks, as well as informal exchanges, e.g., one-on-one feedback and spontaneous coaching (28, 29). Supervisors, positioned at the frontline of the organizational hierarchy, are typically tasked with implementing and reinforcing safety policies, procedures, and behavioral norms (29). The ability to communicate effectively regarding safety is often regarded as an essential competence of supervisors (30). Hofmann and Morgeson (31) emphasized that when workers feel free to communicate safety concerns with supervisors, they develop a strong commitment to safety, leading to fewer accidents. Studies by Laurence (32) and Lingard et al. (16) show that supervisors who engage in regular, open safety communication foster a positive safety climate within workgroups, which further enhances safety performance. Zhang et al. (10) examined the direct effect of SSC on workers' SB and the mediating role of safety climate in this relationship. From a COR perspective, SSC serves as a critical social resource that reduces ambiguity, enhances task competence, and fosters psychological safety. In dynamic and hazardous construction environments, SSC remains an essential contributor to workers' SB.

In contrast to SSC, CSC refers to peer-level safety communication among frontline workers (13, 14). These may take the form of collaborative discussions, mutual reminders, or the spontaneous correction of unsafe practices (16, 33). Though less researched than cross-level communication, peer-level communication has been shown to influence the safety climate of workgroups. Lingard et al. (16) and Pandit et al. (34) demonstrated that denser peer-level communication networks contribute to better hazard recognition and an enhanced safety climate. Despite its importance, studies on peer-level safety communication remain limited. Due to the high interdependence of construction workers tasks, CSC may serve as a stronger predictor of workers' SB. Frontline workers in the workplace often work in close proximity, making peer-to-peer communication particularly important (35). The study by Lingard et al. (16) also demonstrated that strong CSC can enhance workers' safety awareness, which helps prevent accidents and maintain vigilance. Therefore, research on the impact of CSC on SB among construction workers is essential (82). Current research has overlooked the critical role of CSC and lacks sufficient evidence regarding its significant impact on SB.

As indicated above, SSC and CSC as external social resources had a potentially significant impact on SB. Previous studies have largely confirmed the importance of safety communication in shaping safety behavior, yet they often conflate supervisory and coworker communication under a general safety climate construct. This has led to inconsistent findings regarding their specific pathways to SB. While some studies suggest that SSC enhances safety performance via formal policy reinforcement, others argue that CSC, rooted in informal peer interactions, is more effective for real-time safety adjustments in dynamic settings. Thus, the following hypotheses were proposed:

Hypothesis 1 (H1): SSC positively influences workers' SB.
Hypothesis 2 (H2): CSC positively influences workers' SB.

### 2.2 Mediating roles of SKSE, SM, and POS

COR posits that individuals with abundant resources are better equipped to invest in goal-directed behaviors (36). In this study, SKSE, SM, and POS are conceptualized as psychological resources that mediate the relationship between safety communication and SB.

According to job performance theories, individual performance is primarily determined by knowledge and motivation (18, 37). Safety knowledge and motivation as the two determinants when analyzing safety performance (22, 38). Safety knowledge refers to an employee's understanding of safety practices and procedures (39). Employees who lack sufficient safety knowledge may not comply with safety regulations and participate the safety activities well (40). Self-efficacy may be conceptualized as an individual's confidence in their perceived capabilities to successfully execute tasks and attain desired performance outcomes (41). In previous studies, self-efficacy influenced the selection of behavior, as well as the persistence and effort invested in task execution (42). In the realm of knowledge, self-efficacy manifests as an individual's confidence in their ability to utilize their knowledge to support others in completing tasks and enhancing overall work efficiency (43). Unlike general self-efficacy, SKSE refers to the extent to which an individual perceives their safety knowledge as beneficial and effective for both themselves and others (6).

SM refers to “an individual's willingness to exert effort to enact SB and the valence associated with those behaviors” (17). SM reflects an individual's proactive willingness to engage in safety behaviors, driven by the perceived value and importance of safety. Employees who have insufficient SM may not comply with safety regulations and participate in safety activities (40). Many researchers have found that SM is important determinant of SB. For instance, the longitudinal study of Probst and Brubaker (44) revealed that SM played a lagged role in safety compliance. Vinodkumar and Bhasi (22) found that both safety knowledge and SM could significantly predict safety compliance and safety participation. In the context of construction, Guo et al. (18) found that both safety knowledge and SM had a positive effect on safety participation rather than safety compliance.

Psychological ownership has been described as an affective-motivational construct. It is defined as a state in which individuals feel as though the target of ownership is “theirs,” reflecting a subjective sense of possession toward the ownership object (45). In such cases, workers with higher levels of psychological ownership tend to feel a sense of possession over the matter, thereby perceiving it as “my job.” POS embodies a psychological ownership where safety is perceived as a personal responsibility, fostering a sense of duty and emotional investment in maintaining and improving the safety environment (46). Workers with a high POS are more likely to care about the high quality safety processes within their work teams, participate in the continuous improvement of safety management processes, enhance their own SB in hazardous work environments, and help implement safety programs as part of the team or organization. Gagné and Deci (47) proposed that POS is theoretically driven by psychological internalization. Therefore, this study considers POS as an important psychological factor influencing SB.

Within the COR framework, SKSE, SM, and POS are internal psychological resources that mediate the influence of external communication resources on SB (46, 48, 49), played a mediating role in influencing SB. Therefore, the next six hypotheses were proposed as follows:

Hypothesis 3 (H3): SSC indirectly influences workers' SB through increased SKSE.
Hypothesis 4 (H4): CSC indirectly influences workers' SB through increased SKSE.
Hypothesis 5 (H5): SSC indirectly influences workers' SB through increased SM.
Hypothesis 6 (H6): CSC indirectly influences workers' SB through increased SM.
Hypothesis 7 (H7): SSC indirectly influences workers' SB through increased POS.
Hypothesis 8 (H8): CSC indirectly influences workers' SB through increased POS.

### 2.3 Moderating role of WP

WP refers to job-related pressures that exceed an individual's coping capacity, encompassing physical, psychological, and social stressors, which can adversely affect overall wellbeing and mental health (50, 51). The COR theory and the Job Demands–Resources (JD-R) model offer complementary perspectives on how WP influence safety behavior. COR theory emphasizes that resource depletion triggers stress and impairs goal-directed behaviors. The JD-R model posits that excessive job demands (such as high work pressure) deplete these psychological resources, leading to strain and impairing workers' capacity to engage in safe practices (52). The construction industry is widely recognized as a high-demand sector, characterized by long working hours, demanding tasks, and hazardous working environments. Additionally, significant production pressures, such as tight construction schedules and ineffective management, contribute to increased pressure levels among workers (53, 54). Construction workers facing high levels of WP in hazardous environments are likely to experience adverse psychological states, which may impair their cognitive functioning and, in turn, increase the likelihood of engaging in unsafe behaviors (4). In this context, safety communication, originally designed to serve as a job resource for construction workers, may under high WP instead function as an additional burden (83). This may result in decreased self-efficacy, reduced motivation, and a diminished sense of psychological ownership. Based on this, we proposed the following:

Hypothesis 9a (H9a): WP negatively moderates the direct effect of SSC to SB.
Hypothesis 9b (H9b): WP negatively moderates the direct effect of CSC to SB.
Hypothesis 9c (H9c): WP negatively moderates the mediating effect of SSC to SKSE/SM/POS.
Hypothesis 9d (H9d): WP negatively moderates the mediating effect of CSC to SKSE/SM/POS.

### 2.4 Theoretical framework: resource acquisition–resource transformation–behavior model based on COR

A review of the existing literature indicates that research on SSC has primarily emphasized its direct impact on SB, while largely neglecting the underlying internal psychological mechanisms through which SSC influences individual SB. Research on the impact of CSC on SB remains insufficient, with most studies focusing on its influence on safety climate. In the use of mediating variables, this study innovatively adopts SKSE, SM, and POS. This differs from previous studies in the field of SB research, where, for example, safety climate was employed as a mediating variable by Shen et al. (55), Xue et al. (56) and He et al. (57). The inclusion of intrinsic psychological factors as antecedents of external behavior highlights individuals' subjective agency in engaging in SB, particularly in the context of high-risk work environments. Rather than relying on passive interventions, actively promoting SB through psychological engagement may be a more effective strategy.

**Figure 1:**
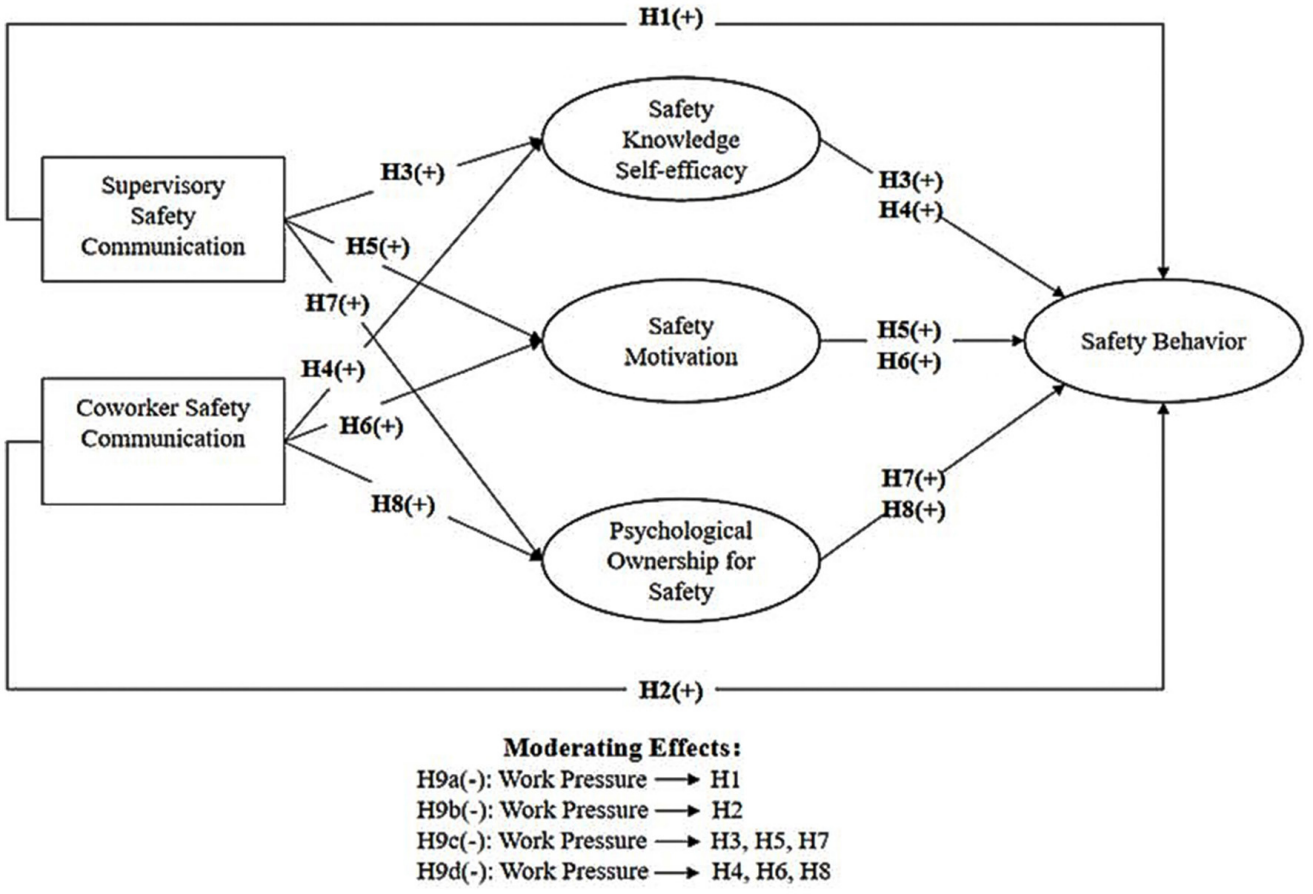
The proposed conceptual model and hypotheses.

This study draws on COR to propose an integrated resource acquisition-resource transformation-behavior (see Figure 1). SSC and CSC are conceptualized as external social resources that enhance workers' internal psychological resources—namely, SKSE, SM, and POS. According to COR theory, individuals strive to accumulate and protect resources not only in isolation but also as part of resource caravans—interconnected sets of resources that travel together. In the construction context, sustained SSC and CSC can act as resource caravan passageways, delivering both informational and emotional resources that help build SKSE, SM, and POS over time. However, under high WP, workers may experience loss spirals, where insufficient resources lead to poor safety outcomes, which in turn further deplete psychological resources and exacerbate pressure.

## 3 Research methods

### 3.1 Data collection

#### 3.1.1 Sample and procedure

A questionnaire survey was conducted in this study to investigate the research objectives. A cross-sectional research design was adopted, collecting data from a population at a specific period, consistent with previous safety compliance and safety participation studies (58–60). Unlike longitudinal studies that track changes over time, this approach provided a snapshot of the target population, suitable given the time and cost constraints of the study. As no comprehensive sampling frame exists for construction workers in China, a non-probability convenience sampling method was employed, consistent with prior studies in this context (61). To mitigate potential biases, data were collected from multiple sites across various project types and locations. Participant eligibility was restricted to construction manual workers directly exposed to on-site hazards, verified through pre-screening. Senior project managers confirmed that the sample's demographic and occupational profile reflected the typical composition of the workforce on large-scale projects in the region. To minimize biases arising from the homogeneity of subjects, context and measurement, several approaches were implemented. First, data were collected from workers at multiple construction sites to reduce location-specific biases. Second, questionnaire items were randomized to prevent participants from inferring the research intent, which could influence responses. Third, the questionnaire used simple, clear language to accommodate workers with lower educational levels, and on-site assistance was provided when needed.

A pilot study involving 15 construction workers and three statistical experts was conducted to evaluate the questionnaire's clarity, relevance, and reliability. Feedback led to the rephrasing of items with complex terminology to enhance comprehensibility. Cronbach's alpha values from the pilot study exceeded 0.70 across all constructs, indicating acceptable reliability prior to main data collection. The questionnaire was then reviewed and approved by the Human Research Ethics Committee to ensure ethical compliance prior to distribution. To ensure sufficient statistical power for hypothesis testing, a total of 425 questionnaires were distributed to frontline construction workers across major infrastructure and building projects in China. After excluding incomplete or inconsistent responses, 359 valid questionnaires were retained, yielding a valid response rate of 84%. This relatively high response rate, compared to similar construction management studies (33, 59), was attributed to the engagement of project managers in facilitating survey distribution and the provision of on-site support. A power analysis conducted using GPower, with an alpha level of 0.05, statistical power of 0.80, and a medium effect size (*f*^2^ = 0.15), confirmed that the sample size of 359 was sufficient to detect significant effects in the proposed model.

Detailed demographic characteristics of the survey respondents are summarized in Table 2. The sample comprised 295 males (82%) and 64 females (18%), reflecting the male-dominated nature of the Chinese construction industry (62). The educational distribution shows that 82 respondents (23%) have primary school education or below, 171 (48%) have completed secondary education, and 106 (29%) have tertiary education or above, indicating a predominance of workers with limited formal education, consistent with the physically demanding nature of construction trades where skills are often acquired on-the-job. The age distribution is concentrated between 20 and 40 years (59%, *n* = 210), with 38% (*n* = 135) having 1–5 years of work experience, 22% (*n* = 78) having 6–15 years, and 16% (*n* = 59) having over 15 years. The sample primarily include workers engaged in infrastructure projects (30%, *n* = 106) and building projects (38%, *n* = 136), with the remaining 32% (*n* = 117) in mixed or other project types. This targeted selection aims to ensure comprehensive coverage of key on-site roles, including reinforcing steel workers (rebar workers), formwork carpenters, concrete workers, electrical and mechanical installation workers, surveyors (layout workers), and general laborers, coordinated through multiple project managers to ensure broad representation across major construction trades. To validate the sample's representativeness, consultations with senior project managers confirmed that the demographic and occupational distribution aligned with the broader workforce patterns in the surveyed projects.

**Table 2:** Demographics of the respondents.

**Background information**	**Categories**	**Frequency**	**Percentage**
Gender	Male	295	82%
	Female	64	18%
Educational level	Primary or below	82	23%
	Secondary	171	48%
	Certificate/diploma	57	16%
	Degree or higher	49	13%
Age	< 20 years	76	21%
	20–29 years	122	34%
	30–39 years	88	25%
	40–49 years	40	11%
	50–60 years	30	8%
	>60 years	3	1%
Project type	Infrastructure	106	30%
	Industrial construction	96	27%
	Building projects	136	38%
	Agricultural/water conservancy	7	2%
	Other	14	3%
Working experience	< 1 years	87	24%
	1–5 years	135	38%
	6–10 years	46	13%
	11–15 years	32	9%
	>15 years	59	16%
Duration on current project	< 3 months	96	27%
	3–6 months	115	32%
	7–10 months	40	11%
	>10 months	108	30%

#### 3.1.2 Measures

The survey questionnaire consisted of two sections. Section A gathered respondents' demographic information, including age, gender, education level, years of experience in the construction industry, and job type. Section B contained construct-specific items, primarily adapted from validated scales in prior research to ensure reliability and validity (63). Each construct was rated using a 5-point Likert scale, ranging from 1 (strongly disagree) to 5 (strongly agree), with participants rating items based on their relevant work experiences at their current worksite. All measurement items underwent a rigorous translation and back-translation process to ensure linguistic and conceptual equivalence. A panel of bilingual experts in construction safety reviewed the translated items for cultural relevance. A pilot test with 15 construction workers further validated item clarity and contextual appropriateness. Additionally, expert consultations were conducted to assess the appropriateness and clarity of the items within the local sociocultural context.

A comprehensive overview of the constructs and their sources is presented in Table 3. SSC was measured using an eight-item scale developed by Huang et al. (15), which was also applied by Zhang et al. (10) to examine the impact of SSC on the safety climate. CSC was assessed through an eleven-item scale proposed by Cong et al. (64). SB was measured following the framework of Neal et al. (38), which divides SB into two dimensions: safety compliance and safety participation. Six items adapted from Neal and Griffin (17) were employed to capture these two aspects. SKSE was measured using a four-item scale adapted from Ishdorj et al. (6), which itself refined earlier scales developed by Zhang and Ng (65) and Kalman (66). SM was assessed using a four-item scale developed by Guo et al. (18), designed to reflect workers' intrinsic motivation toward safety. POS was measured using a four-item scale adapted from Curcuruto et al. (46), focusing on individuals' sense of ownership and responsibility for safety. Finally, WP was evaluated using a four-item scale originally developed by Motowidlo et al. (67), which has been widely applied and validated in occupational stress studies, including recent adaptation for the construction context (68). A pilot test was conducted to assess the questionnaire's format, content, clarity, terminology, and ease of completion. Feedback from statistical experts and pilot test participants was used to refine item wording and structure. Additionally, two independent researchers reviewed the questionnaire to ensure content validity, resolving any discrepancies to minimize errors, bias, and inconsistencies.

**Table 3:** Construct measurement.

**Variables**	**Items**	**Sources**
SSC	SSC1. I feel comfortable discussing safety issues with my supervisor.	(10 , 15 )
	SSC2. I try to avoid talking about safety issues with my supervisor. (Reverse).	
	SSC3. I feel that my supervisor openly accepts ideas for improving safety.	
	SSC4. I am reluctant to discuss safety-related problems with my supervisor. (Reverse)	
	SSC5. I feel that my supervisor encourages open communication about safety.	
	SSC6. Safety information is always brought to my attention by my supervisor.	
	SSC7. My supervisor does not always inform me of current concerns and issues. (Reverse)	
	SSC8. There is good communication here about safety issues which affect me.	
CSC	CSC1. I can freely ask questions to co-workers if I have safety problems.	(64)
	CSC2. When I am not sure about my operation, I will ask co-workers to make sure.	
	CSC3. When I don't understand the safety signs on sites, I will ask co-workers for explanations.	
	CSC4. I find it troublesome to ask co-workers for safety help and explanations. (Reverse)	
	CSC5. Co-workers are willing to answer my safety questions.	
	CSC6. I will tell co-workers how to operate safely if they are working unsafely.	
	CSC7. I will tell the truth when co-workers ask me about safety-related issues.	
	CSC8. Co-workers often remind me to pay attention to safety and help me solve safety issues.	
	CSC9. I will remind co-workers to pay attention to safety and give them safety suggestions.	
	CSC10. I feel comfortable discussing safety issues with co-workers.	
	CSC11. I would like to share my safety operation experience with co-workers.	
	CSC12. When co-workers are discussing safety issues, I will actively participate.	
SB	SB1. I promote the safety program within the organization.	(17)
	SB2. I put in extra effort to improve the safety of the workplace.	
	SB3. I voluntarily carry out tasks or activities that help to improve workplace safety.	
	SB4. I use all the necessary safety equipment to do my job.	
	SB5. I use the correct safety procedures for carrying out my job.	
	SB6. I ensure the highest levels of safety when I carry out my job.	
SKSE	SKSE1. I have sufficient construction safety-related knowledge and experience	(6)
	SKSE2. I have the confidence in my ability to provide safety knowledge that crew members consider useful.	
	SKSE3. I have the experience needed to provide useful safety knowledge for the work crew.	
	SKSE4. I can provide efficient safety knowledge as well as other colleagues.	
SM	SM1. I enjoy working safely on site.	(18)
	SM2. Working safely aligns with my personal values.	
	SM3. I feel bad about myself when I don't work safely.	
	SM4. I feel guilty when I don't work safely.	
POS	POS1: I am personally concerned about worker involvement in programs for safety improvement.	(46)
	POS2: I am personally concerned about stimulating worker initiatives for safety.	
	POS3: I am personally concerned about ensuring personal engagement in safety by every team member.	
	POS4: I am personally concerned about exploring new ways to manage safety in work activities.	
WP	WP1: My work is very stressful.	(67, 68)
	WP2: Few things without pressure in my work.	
	WP3: I feel great pressure for my career.	
	WP4: I often feel rushed to complete my work tasks on time.	

### 3.2 Data analysis

This study first tested the reliability and validity of the questionnaire. Cronbach's alpha values was employed as a key indicator for evaluating internal consistency reliability. When the value is >0.7, the internal consistency reliability is considered acceptable (69). Validity refers to the extent to which a set of observed variables accurately reflects the underlying construct (70). Confirmatory factor analysis (CFA) was conducted using AMOS software to evaluate the measurement model, and the overall validity of the model was assessed based on multiple fit indices. This study employed SPSS software to conduct multiple linear regression analysis for hypothesis testing, which involved three main steps: (1) regressing the mediating variables on the independent variables, (2) regressing the dependent variables on the independent variables, and (3) regressing the dependent variables on both the mediating and independent variables simultaneously (71). To examine the potential moderating effects, interaction terms between the independent variables and the moderator were incorporated into the regression models. In addition, simple slope analyses were conducted, and interaction plots were generated to visually present the nature and direction of the moderation effect.

## 4 Research results

### 4.1 Reliability and validity

The reliability of the scales used in this study was assessed using Cronbach's alpha, which was calculated with SPSS 25.0. The reliability of the questionnaire was considered satisfactory, as the Cronbach's alpha values exceeded the baseline threshold of 0.7, ranging from 0.839 to 0.963 (Table 4). The validity was assessed through CFA, including convergent and discriminant validity. Convergent validity was supported if factor loadings exceeded 0.50, composite reliability (CR) exceeded 0.70, and average variance extracted (AVE) exceeded 0.50. The model in this study demonstrated a good overall fit across multiple indices: χ^2^*/*d*f* = 1.513, goodness-of-fit index (GFI) = 0.904, root mean square error of approximation (RMSEA) = 0.038, Tucker-Lewis index (TLI) = 0.965, comparative fit index (CFI) = 0.968, incremental fit index (IFI) = 0.968, and non-normed fit index (NFI) = 0.911. In addition, the CR values ranged from 0.841 (WP) to 0.963 (CSC and SKSE), and the AVE values ranged from 0.571 (WP) to 0.761 (SB) (Table 4). All of these exceeded the corresponding benchmark values, indicating that the convergent validity of the measurements is satisfactory. Discriminant validity was established when the square root of AVE for each construct exceeded its correlations with other constructs. As indicated in Table 5, the square root of the AVE values for all variables (on the diagonal) is greater than their correlation coefficients with other variables (off the diagonal), demonstrating good discriminant validity. Table 5 also provides descriptive statistics (means and standard deviations) for each variable, along with the initial correlation coefficients, offering a preliminary understanding of the relationships among the variables.

**Table 4:** Measures reliability and validity assessment.

**Constructs and constituent items**	**SFL**	**α**	**AVE**	**CR**
**SSC**				
SSC1	0.796	0.930	0.650	0.930
SSC2	0.774			
SSC3	0.825			
SSC4	0.788			
SSC5	0.827			
SSC6	0.814			
SSC7	0.790			
SSC8	0.833			
**CSC**				
CSC1	0.825	0.963	0.685	0.963
CSC2	0.783			
CSC3	0.795			
CSC4	0.776			
CSC5	0.840			
CSC6	0.854			
CSC7	0.833			
CSC8	0.813			
CSC9	0.821			
CSC10	0.865			
CSC11	0.852			
CSC12	0.862			
**SKSE**				
SKSE1	0.849	0.888	0.684	0.963
SKSE2	0.786			
SKSE3	0.822			
SKSE4	0.807			
**SM**				
SM1	0.847	0.890	0.669	0.890
SM2	0.809			
SM3	0.817			
SM4	0.799			
**POS**				
POS1	0.811	0.905	0.705	0.905
POS2	0.876			
POS3	0.833			
POS4	0.837			
**SB**				
SB1	0.898	0.950	0.761	0.950
SB2	0.879			
SB3	0.889			
SB4	0.867			
SB5	0.844			
SB6	0.855			
**WP**				
WP1	0.766	0.839	0.571	0.841
WP2	0.714			
WP3	0.747			
WP4	0.789			

χ^2^/df = 1.513, GFI = 0.904, RMSEA = 0.038, TLI = 0.965, CFI = 0.968, IFI = 0.968, and NFI = 0.911.

SFL, standardized factor loading; α, Cronbach's alpha coefficient; CR, composite reliability; and AVE, average variance extracted.

**Table 5:** Means, standard deviations, and correlations.

**Variable**	**Mean**	**SD**	**1**	**2**	**3**	**4**	**5**	**6**	**7**
SSC	3.71	0.89	**0.806**						
CSC	3.77	0.95	0.487^*^	**0.828**					
SKSE	3.64	1.05	0.604^**^	0.649^**^	**0.827**				
SM	3.72	1.04	0.648^**^	0.659^**^	0.741^*^	**0.818**			
POS	3.67	1.03	0.521^**^	0.556^**^	0.692^*^	0.717^**^	**0.84**		
SB	3.71	1.11	0.736^**^	0.797^**^	0.718^**^	0.784^**^	0.680^**^	**0.872**	
WP	2.20	0.86	−0.488^*^	−0.454	−0.572^*^	−0.622^*^	−0.551^**^	−0.707^*^	**0.756**

Bold signifies that the number is greater than the off-diagonal correlation.

^*^p < 0.05, ^**^p < 0.01.

### 4.2 Results of regress analysis

Table 6 presents the regression analysis results testing the proposed hypotheses, with *R*^2^ values indicating the proportion of variance explained by each model (ranging from 0.296 to 0.624). It serves as an indicator of the model's explanatory power and predictive utility. A higher *R*^2^ suggested that the predictors account for a substantial amount of the variation in the outcome, while a lower *R*^2^ indicates that other unmeasured factors may be influencing the dependent variable. In terms of *R*^2^ values, all models exhibited relatively good explanatory power. To prevent multicollinearity, the Variance Inflation Factor (VIF) was assessed, with values ranging from 1.27 to 2.94, which are well below the critical threshold of 5. This indicates that there is no significant multicollinearity among the predictors. All models controlled for gender, age, educational level, working type, working experience, project type, and duration on the current project. Key findings are summarized below, with hypothesis testing results in Table 7.

**Table 6:** Results of regression analysis.

**Variables**	**SKSE**		**SM**		**POS**		**SB**					
	**Model 1**	**Model 2**	**Model 3**	**Model 4**	**Model 5**	**Model 6**	**Model 7**	**Model 8**	**Model 9**	**Model 10**	**Model 11**	**Model 12**
**Control variables**												
Gender	0.077	0.090	0.072	0.085	0.028	0.041	0.051	0.030	0.025	0.043	0.022	0.067
Age	0.139	0.063	0.157^*^	0.081	0.191^*^	0.107	0.161^*^	0.124^*^	0.105^*^	0.108^*^	0.082	0.072
Educational level	−0.039	−0.017	0.031	0.053	0.005	0.031	−0.064	−0.053	−0.074	−0.065	−0.068	−0.049
Working type	0.074	0.028	0.106^*^	0.058	0.083	0.032	0.093^*^	0.074	0.055	0.070	0.084	0.046
Working experience	−0.002	0.006	0.072	0.026	−0.030	−0.019	0.035	−0.035	−0.045	−0.027	−0.037	−0.023
Project type	−0.145^*^	−0.124^*^	−0.017	0.007	−0.065	−0.042	−0.043	−0.005	−0.037	−0.025	−0.015	−0.026
Duration on Current Project	0.070	0.073	0.028	0.035	0.002	0.005	0.028	0.010	0.018	0.028	0.014	0.031
**Variables**												
SSC	0.199^**^	0.203^**^	0.222^***^	0.218^***^	0.151^**^	0.165^**^	0.234^***^	0.182^***^	0.156^**^	0.192^***^	0.140^**^	0.201^***^
CSC	0.421^***^	0.371^***^	0.336^***^	0.286^***^	0.333^***^	0.278^***^	0.545^***^	0.434^***^	0.426^***^	0.452^***^	0.375^***^	0.487^***^
SKSE								0.262^***^			0.101^**^	
SM									0.353^***^		0.235^***^	
POS										0.278^***^	0.146^***^	
WP		−0.288^*^		0.309^*^		−0.310^*^						0.352^**^
WP × SSC		−0.277^**^		−0.179^**^		−0.282^**^						−0.193^**^
WP × CSC		0.014		−0.051		0.027						−0.097
*R* ^2^	0.397	0.487	0.445	0.547	0.296	0.414	0.327	0.576	0.595	0.589	0.624	0.540

R-squared is the percentage of the dependent variable's variation that a linear model explains.

^*^p < 0.050; ^**^p < 0.010; ^***^p < 0.001.

**Table 7:** Summary of the results of the hypothesis testing.

**Number**	**Hypotheses**	**Supported or not**
H1	SSC positively influences workers' SB.	Supported
H2	CSC positively influences workers' SB.	Supported
H3	SSC indirectly influences workers' SB through increased SKSE.	Supported
H4	CSC indirectly influences workers' SB through increased SKSE.	Supported
H5	SSC indirectly influences workers' SB through increased SM.	Supported
H6	CSC indirectly influences workers' SB through increased SM.	Supported
H7	SSC indirectly influences workers' SB through increased POS.	Supported
H8	CSC indirectly influences workers' SB through increased POS.	Supported
H9a	WP negatively moderates the direct effect of SSC to SB.	Supported
H9b	WP negatively moderates the direct effect of CSC to SB.	Not supported
H9c	WP negatively moderates the mediating effect of SSC to SKSE/SM/POS.	Supported
H9d	WP negatively moderates the mediating effect of CSC to SKSE/SM/POS.	Not supported

Model 7 shows that SSC (β = 0.234, *p* < 0.001) and CSC (β = 0.545, *p* < 0.001) significantly predict SB, supporting H1 and H2. For mediation, Model 1 shows SSC (β = 0.199, *p* < 0.01) and CSC (β = 0.421, *p* < 0.001) have significant positive effects on SKSE. When SKSE is added in Model 8, the positive effects of SSC (β = 0.182, *p* < 0.001) and CSC (β = 0.434, *p* < 0.001) on SB are attenuated, indicating that SKSE mediates the relationship between SSC/CSC on SB, thus supporting H3 and H4. Similarly, Model 3 shows that SSC (β = 0.222, *p* < 0.001) and CSC (β = 0.336, *p* < 0.001) significantly and positively influence SM. After including SM in Model 9, the effects of SSC (β = 0.156, *p* < 0.01) and CSC (β = 0.426, *p* < 0.001) on SB are weakened, suggesting that SM serves as a mediator in the relationship between SSC/CSC and SB, thereby supporting H5 and H6. Finally, Model 5 demonstrates that SSC (β = 0.151, *p* < 0.01) and CSC (β = 0.333, *p* < 0.001) have significant positive effects on POS. When POS is added in Model 10, the positive effects of SSC (β = 0.192, *p* < 0.001) and CSC (β = 0.452, *p* < 0.001) on SB are reduced, indicating that POS mediates the relationship between SSC/CSC and SB, thus supporting H7 and H8. Besides, Model 11 further demonstrates the mediating effects of SKSE (β = 0.101, *p* < 0.01), SM (β = 0.235, *p* < 0.001), and POS (β = 0.146, *p* < 0.001), indicating that all three serve as partial mediators in the relationship. Mediation analyses followed Baron and Kenny (71) three-step criteria.

**Figure 2:**
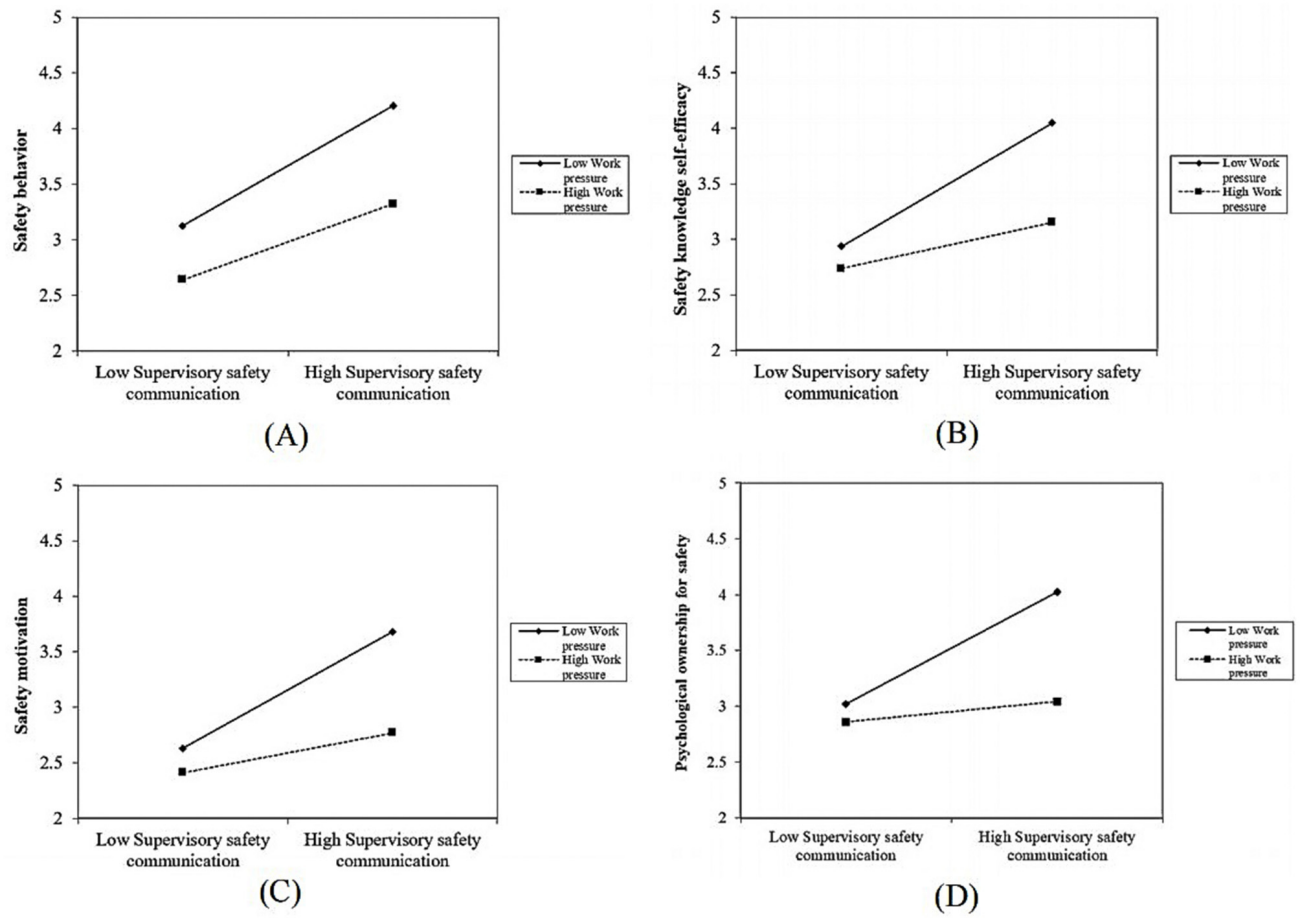
**(A–D)** Slope plot of the moderation effect.

For moderation, Models 2, 4, and 6 show that WP negatively moderates the relationships between SSC and SKSE (β = −0.277, *p* < 0.01), SM (β = −0.179, *p* < 0.01), and POS (β = −0.282, *p* < 0.01), supporting H9c. However, WP does not significantly moderate the CSC relationships with SKSE, SM, and POS (β = −0.051 to 0.027, *p* > 0.05), failing to support H9d. Similarly, Model 12 indicates that WP negatively moderates the SSC and SB relationship (β = −0.193, *p* < 0.01), supporting H9a, but not the CSC and SB relationship (β = −0.097, *p* > 0.05), failing to support H9b. The non-significant moderation for CSC may reflect contextual factors, such as varying coworker dynamics, warranting further exploration. The potential reasons for this phenomenon will be further discussed in the following section. No suppression effects were observed in the mediation pathways, indicating consistent directional relationships and significant indirect effects. Based on the study by Cohen et al. (72), this research plotted the interaction effects as illustrated in Figure 2, demonstrating how the relationships between SSC and SB, SKSE, SM, and POS vary under conditions of high and low WP (*p* < 0.05). The simple slope analysis further confirmed the moderating effect of WP, indicating that the influence of the SSC on the SB, SKSE, SM, and POS varied significantly across different levels of WP. As shown in the figure, POS is more susceptible to the moderating effect of WP.

## 5 Discussion

### 5.1 Key findings

#### 5.1.1 Direct effects of SSC and CSC on SB

This study supported H1 and H2, showing that both SSC and CSC had significant positive effects on SB. This reinforces the crucial role of supervisory and peer-to-peer safety communication in shaping workers' behavioral outcomes on construction sites. The results echo prior research suggesting that effective safety communication enhances workers' safety participation and compliance (3, 73). The finding that SSC significantly influences SB is consistent with the study of Zhang et al. (10), which emphasized that supervisors, beyond effectively conveying safety information, must also actively listen to workers' safety concerns and ideas. SSC as a relatively formal mode of communication, helps establish clear safety expectations, define responsibilities, and reinforce organizational safety standards (15). In contrast, CSC represents informal and horizontal exchanges that occur naturally among workers. Such peer-to-peer communication plays a complementary role by reinforcing safety norms, bridging communication gaps left by formal systems, and providing real-time, context-relevant feedback in a more relatable and trust-based manner (16, 64).

Notably, the standardized effect of CSC (β = 0.545, *p* < 0.001) was more than twice that of SSC (β = 0.234, *p* < 0.001), suggesting that coworker-based communication may exert a more substantial influence on workers' actual safety behaviors. The observed stronger influence of CSC may partially reflect characteristics of collectivist and high-context work environments where peer influence is pivotal. Nevertheless, as we did not directly measure cultural orientation, this interpretation remains tentative and warrants further cross-cultural comparative research. Under the high-pressure and dynamic conditions of construction sites, coworker guidance may be perceived as more direct, relevant, and trustworthy than formal communication from management (33). Moreover, frontline workers' tasks are often highly interdependent, and the hazards they face are frequently situational in nature (35). Coworker reminders or real-time warnings (such as a coworker pointing out a tripping hazard) can trigger more immediate behavioral responses than periodic briefings or posted signs. This immediacy may help explain the relatively stronger effect of CSC on frontline SB observed in this study.

SSC, as a top-down, formal mechanism, establishes clear expectations and aligns workers with organizational safety standards (15). However, its influence may be more limited by hierarchical distance or perceptions of formality. In contrast, CSC naturally occurs among workers within shared routines, providing context-relevant safety advice in a more relatable and trust-based format (16). While SSC has been widely researched, CSC remains under-theorized. The quality and frequency of coworker interactions—especially those based on egalitarian principles—may play a critical yet underexplored role in shaping perceptions of fairness, group cohesion, and ultimately SB (74). This study addresses this gap by demonstrating the distinct and robust impact of CSC, thereby emphasizing the value of informal, coworker-driven safety dynamics in high-risk environments. This suggested that safety interventions should not only empower supervisors but also cultivate a culture of coworker safety leadership and mutual monitoring.

#### 5.1.2 Mediating role of SKSE, SM, and POS

This study also confirmed the mediating roles of SKSE, SM, and POS in the relationships between both SSC and CSC with SB, supporting H3 through H8. These mediating pathways highlight that communication—whether SSC or CSC—does not exert influence on SB in a vacuum, but rather through shaping critical individual psychological states. Specifically, SSC and CSC were found to significantly enhance SKSE (β = 0.199–0.421), SM (β = 0.222–0.336), and POS (β = 0.151–0.333), each of which, in turn, significantly predicted SB (SKSE: β = 0.101; SM: β = 0.235; POS: β = 0.146). This suggests that effective safety communication builds workers' confidence in their ability to understand and apply safety knowledge, enhances their intrinsic motivation to engage in safe practices, and fosters a stronger psychological ownership toward the shared goal of workplace safety (49, 75, 76). These internal psychological resources act as bridges, translating external communication efforts into internalized beliefs and attitudes, which ultimately manifest in observable SB (84). This result is largely consistent with findings from previous studies, which have similarly emphasized the role of individual psychological in translating safety communication into behavioral outcomes (5, 77).

This finding echoes prior research that emphasizes the importance of self-efficacy and motivation in promoting SB (18, 77), and contributes novel insights by demonstrating the mediating role of POS. While POS has been examined in organizational commitment and voice behavior domains (78, 79), its role in safety contexts has been underexplored. This study extends the POS literature by confirming its predictive power for SB, consistent with recent findings by Kuang et al. (23), thereby reinforcing its cross-contextual applicability. By integrating POS into the safety communication framework, this study contributes a richer understanding of how ownership-based psychological states foster individual safety responsibility and behavioral engagement.

Although all three mediators promote the relationship between SSC/CSC and SB, the strength of their respective pathways differs, suggesting that their underlying mechanisms may be conceptually distinct. SKSE reflects a cognitive belief in one's ability to perform work safely (6); SM represents an emotional willingness to engage in safety practices (17); and POS indicates a psychological investment in a safe work environment (46). In Model 11, SM exhibits a particularly strong effect (β = 0.235, *p* < 0.001), indicating that motivational states may play the most immediate role in shaping on-site SB—especially in dynamic and high-risk construction settings where rapid and proactive actions are often required.

Notably, while SSC and CSC influenced all three mediators, CSC appeared to exert relatively stronger effects on SKSE, SM and POS. This phenomenon may be attributed to the relational nature and egalitarian essence of coworker communication (6). Coworker interactions typically lack clear power hierarchies, and such decentralized communication helps create an open and trusting environment in which workers feel more comfortable expressing their genuine thoughts, especially when it comes to safety issues. This type of supportive interaction also provides stable internal psychological resources that sustain their ongoing engagement in SB.

Although SKSE, SM, and POS were examined as parallel mediators in this study, it is plausible that they are not entirely independent psychological processes. For instance, SKSE may serve as a cognitive foundation for SM—workers who believe in their ability to perform tasks safely are more likely to be intrinsically motivated to do so. Likewise, heightened motivation may reinforce a sense of psychological ownership, as motivated individuals tend to internalize collective goals. These interdependencies suggest that psychological mechanisms linking safety communication to behavior may operate in a sequential or reinforcing manner, rather than in isolation. Future research could empirically test these layered mediation structures using longitudinal designs or advanced modeling techniques such as serial mediation or latent profile analysis.

#### 5.1.3 Moderating role of WP

Finally, the results reveal important boundary conditions by showing that the effectiveness of SSC is significantly moderated by WP, while CSC remains unaffected. Specifically, WP negatively moderated the relationships between SSC and SKSE, SM, POS, and SB, supporting H9a and H9c. However, its moderating effect on the corresponding CSC pathways was non-significant, failing to support H9b and H9d.

This asymmetry suggests that SSC is more vulnerable to situational stressors. Under high WP, workers face cognitive overload due to time pressure, task complexity, and competing demands between safety and productivity. In such conditions, they may lack the cognitive and emotional resources to fully process and respond to SSC, leading to disengagement or avoidance (80). Additionally, as supervisors are often perceived as the source of WP, SSC may be met with skepticism or interpreted as contradictory, further diminishing its impact (81). By contrast, CSC appears more resilient. As a horizontal form of communication, it is perceived as more empathetic, organically embedded in work routines, and less cognitively demanding. It may reflect shared practical knowledge and mutual concern, making it more effective under stress. Moreover, coworker support may serve as a coping resource in high-pressure environments, helping to maintain SB despite organizational strain.

### 5.2 Theoretical contributions

This study makes several important theoretical contributions to the safety communication and construction management literature. Firstly, it empirically distinguishes between the roles of SSC and CSC in influencing SB, emphasizing CSC as a relatively stronger predictor. While previous research has focused primarily on SSC, this study adds to the growing recognition that coworker-based communication is not only complementary but also independently effective, especially in collectivist, high-context work cultures. By analyzing both SSC and CSC in the same analytical framework, they are examined at the same level, effectively emphasizing the equal importance of both. Secondly, by combining three psychological mediators (SKSE, SM, and POS), this study deepens the understanding of how safety communication translates into behavioral outcomes. It extends the COR perspective by showing that external SB is internalized through self-efficacy, motivation, and psychological ownership, which are key intra-individual resources, and notably, the inclusion of POS as a mediator is a novel contribution that has rarely been explored in construction safety research. This broadens the theoretical perspective for understanding SB. Thirdly, the study reveals that at high WP erodes external social and internal psychological resources. By identifying WP as a negative moderator that selectively weakens the SSC pathway rather than the CSC, the study introduces a nuanced view of how situational demands interact with communication channels. This finding contributes to safe communication theory by emphasizing that SSC effectiveness is more susceptible to cognitive overload and stress, whereas coworker-based CSC may be more cognitively resilient, especially in dynamic, high-stakes environments.

### 5.3 Practical implications

The findings offer several actionable insights for construction managers, safety professionals, and policymakers seeking to improve SB on worksites. Given the strong and consistent impact of CSC on SB (even at high WP), relevant organizations should invest in facilitating colleague-based safety discussions. This could include the development of a coworker mentoring program, crew-based conversations, or a safety buddy system where safety conversations become part of the daily routine. Also strengthening the CSC communication network, the CSC can act as a buffer against the negative effects of work stress and its role should be recognized and supported in the organization's safety strategy. Since the impact of SSC on SB is significantly diminished at high wp, supervisors should be trained not only on how to communicate safety information, but also when and under what conditions. Embedding safety communication in daily workflows, ensuring that it is bi-directional rather than command-driven, and combining it with visible management support can help to mitigate the adverse effects of high cognitive load. At the same time, the organization should monitor and manage stress-inducing workload conditions and ensure that safety messages are not perceived as conflicting with production goals. While this study emphasizes the psychological mechanisms through which safety communication influences behavior, it is critical to recognize that such communication does not occur in a vacuum. Organizational structures—such as rigid hierarchies or fragmented subcontracting systems—can hinder open communication and dilute the influence of both SSC and CSC. Therefore, interventions should be embedded within supportive structures that empower both supervisors and workers to engage in two-way communication. Moreover, leadership styles play a vital role in shaping communication climates. For instance, transformational leadership may amplify the effectiveness of SSC by fostering trust and encouraging voice behavior, whereas authoritarian leadership may suppress psychological ownership and self-efficacy. Finally, the effectiveness of any safety initiative is contingent upon broader labor policies, including contractual stability, worker representation mechanisms, and safety enforcement practices. We recommend that future interventions be co-designed with organizational stakeholders and policy actors to ensure alignment with existing structural realities and to enhance sustainability.

## 6 Conclusions

This study contributes to the growing body of research on construction safety by empirically examining how SSC and CSC influence workers' SB through key psychological mechanisms, including SKSE, SM, and POS. The results highlight the distinct yet complementary roles of SSC and CSC. While both SSC and CSC significantly enhance SB, SSC was found to be more susceptible to the negative influence of WP, likely due to its association with performance demands imposed by supervisors. In contrast, CSC remained stable under pressure, underscoring its value as a consistent and trusted safety resource. Furthermore, the mediating roles of SKSE, SM, and POS confirm that internal psychological states are critical pathways through which safety messages are translated into behavioral outcomes. This study extends the application of COR theory by demonstrating how safety communication functions as a social resource that builds psychological capacities conducive to safety behavior in construction settings. While our findings enrich the understanding of communication's role within the COR framework, we recognize that this constitutes an incremental rather than a transformative theoretical contribution.

Despite its contributions, this study is not without limitations. First, the use of convenience sampling introduces sampling bias, which may limit the representativeness of the findings beyond the sampled projects. Second, the reliance on self-reported data for all key variables introduces the potential for common method variance (CMV), which may inflate or deflate relationships between variables due to shared method biases, such as response tendencies or social desirability. Third, the use of a cross-sectional design restricts our ability to make causal inferences regarding the mediating and moderating mechanisms tested. While statistical methods offer preliminary evidence, future research should employ longitudinal or experimental designs to more rigorously establish causality. Last, while our model includes key psychological mediators, we did not control for other organizational variables such as safety climate, leadership support, or formal safety policies, which may also impact safety behavior. Omitted variable bias could arise from unmeasured individual or contextual factors that were not included in the model. Addressing these limitations in future research through probabilistic sampling, multi-source data, and multilevel modeling would enhance the robustness of the conclusions.

## Data Availability

The raw data supporting the conclusions of this article will be made available by the authors, without undue reservation.
